# Factors associated with lower quarter performance-based balance and strength tests: a cross-sectional analysis from the project baseline health study

**DOI:** 10.3389/fspor.2024.1393332

**Published:** 2024-07-15

**Authors:** Kenneth A. Taylor, Megan K. Carroll, Sarah A. Short, Bettia E. Celestin, Adam Gilbertson, Christoph B. Olivier, Francois Haddad, Nicholas Cauwenberghs

**Affiliations:** ^1^ Orthopaedic Surgery, Duke University School of Medicine, Durham, NC, United States; ^2^ Duke Clinical Research Institute, Duke University School of Medicine, Durham, NC, United States; ^3^ Verily Life Sciences, San Francisco, CA, United States; ^4^ Allergy and Immunology, Stanford University School of Medicine, Stanford, CA, United States; ^5^ Durham Veterans Affairs (VA) Healthcare System, Durham, NC, United States; ^6^ Department of Cardiology and Angiology, University Heart Center Freiburg-Bad Krozingen, Faculty of Medicine, University of Freiburg, Freiburg, Germany; ^7^ Division of Cardiovascular Medicine, Stanford University School of Medicine, Stanford, CA, United States; ^8^ Stanford Cardiovascular Institute, Stanford University School of Medicine, Stanford, CA, United States; ^9^ Stanford Diabetes Research Center, Stanford University School of Medicine, Stanford, CA, United States; ^10^ Department of Cardiovascular Sciences, University of Leuven, Leuven, Belgium

**Keywords:** project baseline health study, biomarkers, physical functional performance, single-legged balance test, sitting-rising test, 30-second chair-stand test, community-dwelling adults

## Abstract

**Objectives:**

Physical performance tests are predictive of mortality and may screen for certain health conditions (e.g., sarcopenia); however, their diagnostic and/or prognostic value has primarily been studied in age-limited or disease-specific cohorts. Our objective was to identify the most salient characteristics associated with three lower quarter balance and strength tests in a cohort of community-dwelling adults.

**Methods:**

We applied a stacked elastic net approach on detailed data on sociodemographic, health and health-related behaviors, and biomarker data from the first visit of the Project Baseline Health Study (*N* = 2,502) to determine which variables were most associated with three physical performance measures: single-legged balance test (SLBT), sitting-rising test (SRT), and 30-second chair-stand test (30CST). Analyses were stratified by age (<65 and ≥65).

**Results:**

Female sex, Black or African American race, lower educational attainment, and health conditions such as non-alcoholic fatty liver disease and cardiovascular conditions (e.g., hypertension) were consistently associated with worse performance across all three tests. Several other health conditions were associated with either better or worse test performance, depending on age group and test. C-reactive protein was the only laboratory value associated with performance across age and test groups with some consistency.

**Conclusions:**

Our results highlighted previously identified and several novel salient factors associated with performance on the SLBT, SRT, and 30CST. These tests could represent affordable, noninvasive biomarkers of prevalent and/or future disease in adult individuals; future research should validate these findings.

**Clinical Trial Registration:**

ClinicalTrials.gov, identifier NCT03154346, registered on May 15, 2017.

## Introduction

A person's ability to perform daily living activities—physical function—can worsen by disease or injury and is affected by clinical and sociodemographic factors associated with unfavorable health-related behavior (1). Declines in physical function are often subtle and unnoticed until they reach a stage at which interventions may be less effective, hence the importance of identifying subclinical declines in a timely fashion (2–5).

Standardized clinical tests serve this purpose, enabling affordable and objective quantification of different aspects of physical function, including control of static posture and balance, musculoskeletal fitness and strength, and overall mobility. Several physical function tests can be indicators of prevalent health conditions and predictive of downstream health outcomes and all-cause mortality, particularly in older individuals (6–15).

To date, relationships between physical performance tests and specific clinical characteristics have primarily been studied in age-limited or diagnosis-specific cohorts, which are inherently limited to identify wide range relationships (16). Data from broadly-defined cohorts could reveal novel and comprehensive associations between physical function tests and individual characteristics. Therefore, we aimed to identify the most salient characteristics associated with the three lower quarter physical performance tests (single-legged balance [SLBT], sitting-rising [SRT], and 30-s chair-stand [30CST]), from sociodemographic and health features as recorded in a deeply phenotyped cohort of community-dwelling adults, stratified by age (≥65 years-old and <65 years-old).

## Methods

### Project baseline health study

In this study, we used data from The Project Baseline Health Study (PBHS). The PBHS is a multicenter longitudinal cohort study of adults in the United States (ClinicalTrials.gov identifier NCT03154346), approved by both a central Institutional Review Board (the WCG IRB; approval tracking number 20170163, work order number 1-1506365-1) and IRBs at each of the participating institutions: Stanford University (Palo Alto, CA), Duke University (Durham, NC; Kannapolis, NC), and the California Health and Longevity Institute (CHLI; Los Angeles, CA). Participants' deep phenotyping included data on demographic characteristics, socioeconomic status-related health behaviors, medical conditions, symptoms, and laboratory-collected biomarkers in addition to physical function markers and a range of patient-reported outcome measures. Full description of study procedures and design, inclusion and exclusion criteria, and IRB approval have been previously reported (17, 18).

This study used cross-sectional data from the first in-person study collection time point; self-reported data were either recorded in-person with assistance from study personnel, or collected via remote application. We limited data that were only collected remotely to participant responses within 200 days of participants' initial in-person data collection.

### Physical performance measures

#### Single-legged balance test

The SLBT assesses static postural and balance control with moderate-to-excellent test-retest reliability and excellent inter-rater reliability (19, 20). To perform the SLBT, participants were instructed to place their hands on their hips with their eyes open while focusing on a point straight ahead and stand on one leg while raising the other. The time was recorded from when one foot was lifted from the floor until whichever occurred first: the lifted foot touched the stance leg or the ground, the stance foot moved on the floor, either hand left the hips, or 60 s had passed. The test was performed on each leg one time and the SLBT time recorded was the mean of the left and right leg balance durations. Poor SLBT performance may help in predicting falls (21, 22) and inability to achieve at least 10 s has been linked to all-cause mortality (14).

#### Sitting-rising test

The SRT was developed to assess muscle strength and power, flexibility, and balance components of physical fitness through evaluating an individual's ability to transfer from standing to sitting on the floor and then rise from the floor back to standing (23). The test has also been shown to predict all-cause mortality in a cohort of adults aged 51–80 years-old (15). To perform the SRT, participants were placed on a non-slippery flat surface in a minimum space of 2 × 2 m without wearing shoes. The basic movements of the test were explained to the participant before the participants were instructed, “without worrying about the speed of movement, try to sit then to rise from the floor using the minimum support that you believe is needed.” The test was scored based on performance, with a maximum of 10 points—5 points for transferring from standing to sitting on floor and 5 points transferring from sitting on the floor back to standing. Each participant started with a score of 5 for each movement from which 1.0 point was deducted for each time a hand, forearm, knee, side of the leg (but not the sides of the feet), or hand on the knee was used and 0.5 points deducted for each time a partial loss of balance was observed. Participants who did not obtain a full score on either the sitting or rising portions of the test were provided additional advice or demonstration of the action by site staff and allowed to perform additional attempts to improve their scores. Regardless of the number of attempts performed, only the best scores for each sitting and rising attempt were recorded. Despite the rater-based scoring, SRT has shown high inter-rater reliability (24).

#### 30-second chair stand test

The 30CST is a measure of functional lower extremity strength with excellent criterion, interrater, and test-retest reliability (25). To perform the 30CST, participants were positioned in a 17-in. high straight-backed armless chair and instructed to keep their feet flat on the floor and arms crossed over their chest during the test. Upon hearing “go” participants were instructed to rise to a full standing position and then back down to sitting for 30 s. The test is scored by recording the number of full stands completed in 30 s. Being >50% to a full standing position at the end of the 30 s was counted as a full stand. Since the test is scored using stand count, the 30CST does not have the same floor effect as other standing tests that are scored by recording time-to-completion for a specific number of stands (e.g., 5-time sit-to-stand) (26).

### Other variables

Data collected from PHBS participants has been previously reported (17). We excluded variables with age-specific prevalence ≤1% from regression analyses. In participants aged <65 years, this removed 13 medical conditions from the list of candidate variables: atrial fibrillation, benign prostate hyperplasia, prostate cancer, breast cancer, hepatitis B, myocardial infarction, macular degeneration, melanoma, pulmonary embolism/deep vein thrombosis, peripheral vascular disease, stroke, transient ischemic attack, and goiter. In participants aged ≥65 years, we removed 6 conditions: bipolar disorder, type I diabetes mellitus, drug abuse, fibromyalgia, hepatitis C, and posttraumatic stress disorder. We applied Box-Cox transformations to each laboratory value to approximate Gaussian distributions. We present an ascertainment and definition summary of eligible variables used in our analyses in the Supplementary Material.

### Statistical analysis

We summarized clinical and demographic characteristics for participants with completed physical performance tests. All analyses were stratified by age and models were estimated separately for younger (<65 years) and older (≥65 years) participants. The SLBT was dichotomized for adults aged <65 years as either achieving the maximum time (60 s) or not (<60 s) based on the high proportion of individuals in this subgroup hitting the 60-s ceiling. SLBT was kept as continuous for adults aged ≥65 years-old, which had a wider distribution in this subgroup. The SRT and 30CST did not demonstrate similar ceiling effects and were left as continuous variables across age subgroups. We used multiple imputation by chained equations to estimate values for missing data and fit regression models using elastic net (ENET) regularization methods. We employed ENET using a stacked objective function (sENET) with 5-fold cross-validation to penalize and select regression coefficients modeling each physical performance measure (27, 28). A more detailed description of the sENET methods we used can be found in the Supplementary Material. We performed a sensitivity analysis that added self-reported symptoms to the list of eligible covariate candidates. Additional details and reasoning are presented in the Supplementary Material.

This report follows STROBE guidelines and complies with the STROBE cohort checklist (29).

## Results

### Participants

**Figure 1:**
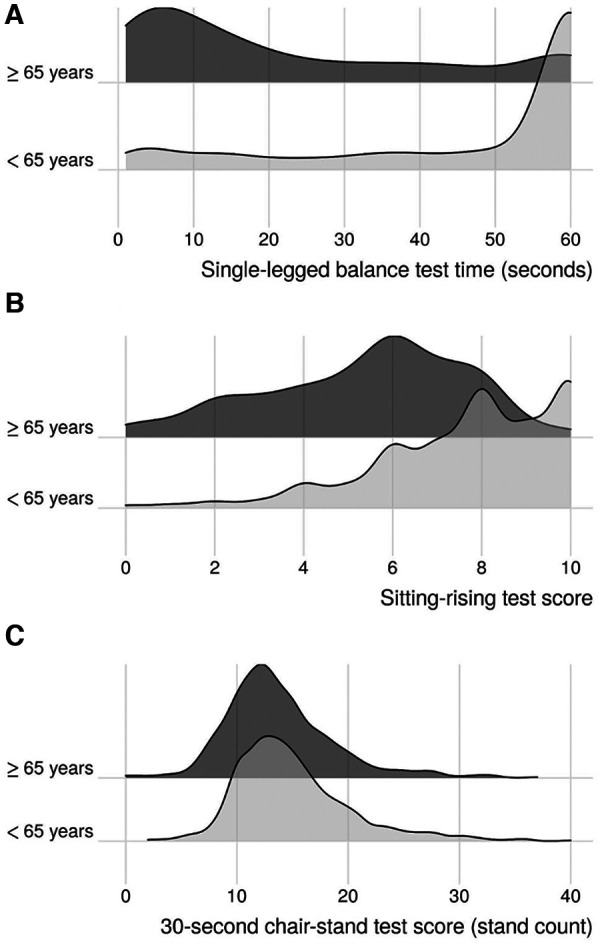
Kernel density ridgeline plots of age-stratified physical performance results. (**A**) Single-legged balance test; (**B**): sitting-rising test; (**C**): 30-s chair-stand test.

Of the 2,502 participants in the PBHS cohort, 2,339 (93.5%), 2,198 (87.9%), and 2,410 (96.3%) completed SLBT, SRT, and 30CST at the initial visit, respectively. Of these, 23.6% (SLBT), 22.2% (SRT), and 23.0% (30CST) were aged ≥65 years. For participants aged <65 years, mean (SD) physical performance test scores were 47 (20) seconds for SLBT, 7.7 (2.1) points for SRT, and 15 (5.2) stands for 30CST. For participants ≥65 years of age, mean (SD) test scores were 23 (20) seconds, 5.4 (2.3) points, and 14 (5.0) stands for SLBT, SRT, and 30CST, respectively (Figure 1 and Table 1).

**Table 1:** Summary of age-stratified demographic characteristics by lower quarter physical performance test.

Demographics	Single leg balance test		Sitting rising test		30-s chair-stand test	
	<65 years (*N* = 1,787)^a^	≥65 years (*N* = 552)^a^	<65 years (*N* = 1,711)^a^	≥65 years (*N* = 487)^a^	<65 years (*N* = 1,857)^a^	≥65 years (*N* = 553)^a^
Age, years	43.1 (32.0, 54.3)	72.3 (69.1, 76.4)	42.5 (31.8, 53.8)	72.2 (69.1, 76.3)	42.8 (31.9, 54.2)	72.3 (69.0, 76.4)
Female	1,014 (56.7)	282 (51.1)	963 (56.3)	244 (50.1)	1,033 (55.6)	281 (50.8)
Hispanic ethnicity	256 (14.3)	15 (2.7)	249 (14.6)	12 (2.5)	264 (14.2)	15 (2.7)
Race						
White	1,016 (56.9)	473 (85.7)	979 (57.2)	415 (85.2)	1,067 (57.5)	474 (85.7)
Black or African American	329 (18.4)	42 (7.6)	303 (17.7)	38 (7.8)	332 (17.9)	41 (7.4)
Asian	214 (12.0)	27 (4.9)	209 (12.2)	26 (5.3)	224 (12.1)	28 (5.1)
Native Hawaiian or other Pacific Islander	23 (1.3)	2 (0.4)	22 (1.3)	2 (0.4)	25 (1.3)	2 (0.4)
American Indian or Alaska Native	24 (1.3)	3 (0.5)	23 (1.3)	3 (0.6)	24 (1.3)	3 (0.5)
Other	181 (10.1)	5 (0.9)	175 (10.2)	3 (0.6)	184 (9.9)	5 (0.9)
Site						
Los Angeles	365 (20.4)	38 (6.9)	348 (20.3)	36 (7.4)	422 (22.7)	40 (7.2)
Durham	387 (21.7)	63 (11.4)	367 (21.4)	53 (10.9)	398 (21.4)	61 (11.0)
Kannapolis	360 (20.1)	143 (25.9)	329 (19.2)	120 (24.6)	361 (19.4)	142 (25.7)
Palo Alto	675 (37.8)	308 (55.8)	667 (39.0)	278 (57.1)	676 (36.4)	310 (56.1)

^a^ Median (IQR); *n* (%).

### Main results

To focus results and avoid reporting on variables with small coefficients in the final models, we present the top 20 candidate characteristics from sENET results. Models for single-legged balance and sitting-rising test scores were optimized to LASSO regressions (*ɑ* = 1), while models for 30CST were optimized to ENET regression (*ɑ* = 0.5).

#### Single-legged balance test

**Figure 2:**
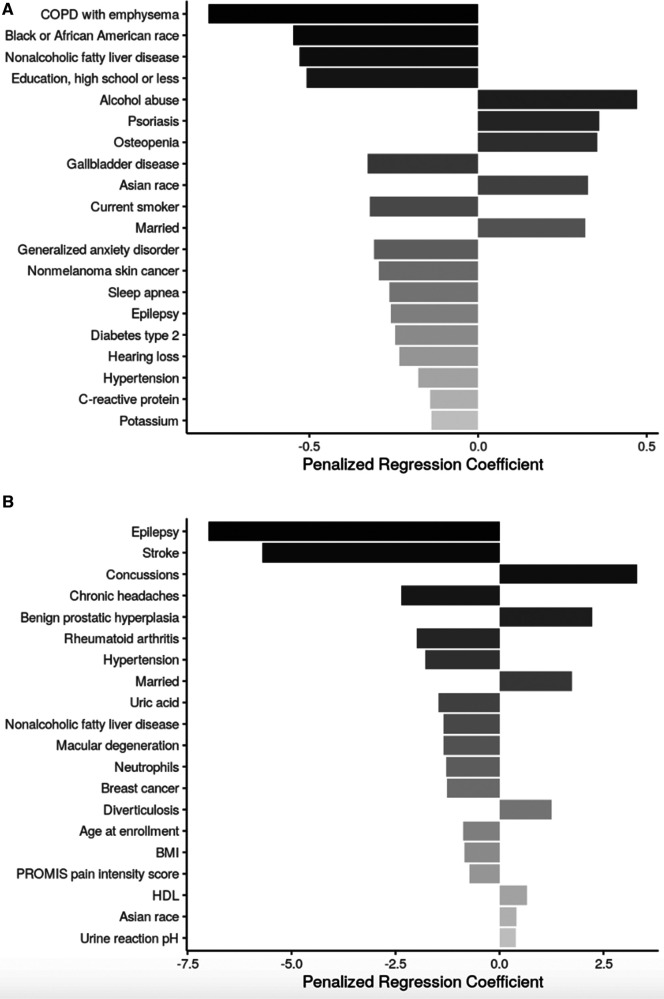
Top 20 regression coefficients of features selected from sENET regression model for single legged balance test. (**A**) Participants <65 years of age (*ɑ* = 1, *λ* = 0.0025); (**B**): participants ≥65 years of age (*ɑ* = 1, *λ* = 0.334). PTSD, posttraumatic stress disorder; BMI, body mass index; HDL, high density lipoprotein.

Across both age groups, sociodemographic factors positively associated with SLBT performance included identifying as Asian or being married. In participants aged <65, identifying as Black or African American or having high school or less as highest educational attainment was also associated with lower odds of achieving the 60-second SLBT ceiling. Among those >65 years-old, continuous age remained an important factor negatively associated with SLBT performance (Figures 2A,B).

Health conditions and behaviors that were associated with worse SLBT performance across both age groups were non-alcoholic fatty liver disease (NAFLD), hypertension, and epilepsy. In adults <65 years-old, chronic obstructive pulmonary disease (COPD) with emphysema, gallbladder disease, being a current smoker, generalized anxiety disorder, and nonmelanoma skin cancer, sleep apnea, and type II diabetes were among the factors associated with lower odds of achieving the 60-s SLBT ceiling while alcohol abuse, psoriasis, and osteopenia were among those associated with increased odds of high SLBT performance. Among adults aged ≥65 years, stroke, chronic headaches, rheumatoid arthritis, macular degeneration, and breast cancer were all associated with lower SLBT times while concussions, benign prostatic hyperplasia, and diverticulosis were associated with higher SLBT times.

Biomarkers associated with SLBT performance in adults <65 years-old included C-reactive protein (CRP; associated with decreased odds of achieving 60 s). In adults aged ≥65 years, body mass index (BMI), uric acid, and neutrophil levels were negatively associated with SLBT time, while high-density lipoprotein (HDL) and urine pH levels were positively associated with SLBT time.

#### Sitting-rising test

**Figure 3:**
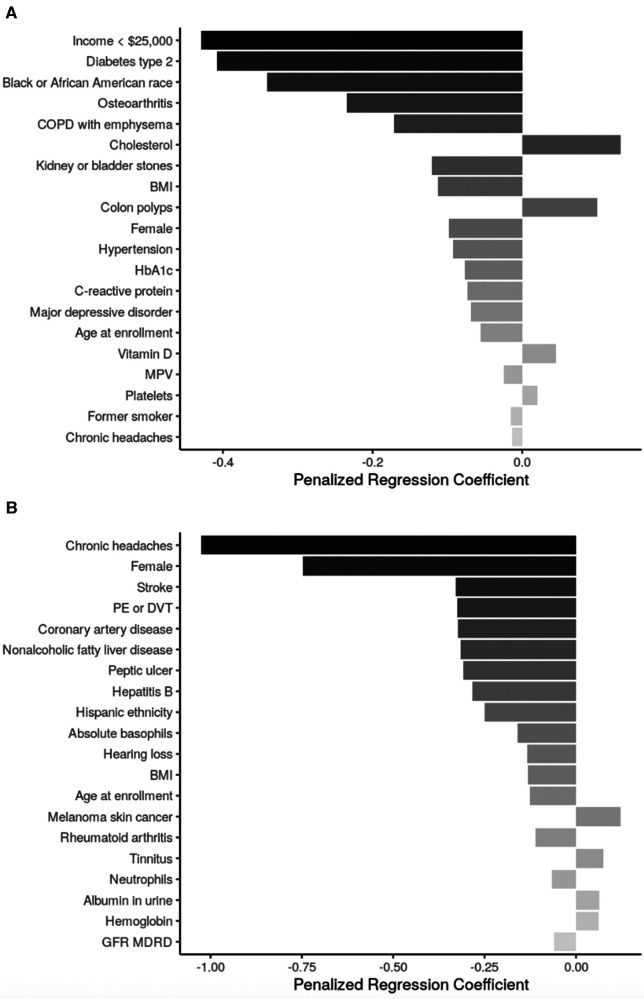
Top 20 regression coefficients of features selected from sENET regression model for sitting rising score. (**A**) Participants <65 years of age (*ɑ* = 1, *λ* = 0.023); (**B**): participants ≥65 years of age (*ɑ* = 1, *λ* = 0.038). BMI, body mass index; HbA1c, hemoglobin A1c; MPV = ; PE, pulmonary embolism; DVT, deep venous thrombosis; GFR MDRD, estimated glomerular filtration rate, modification of diet in renal disease study equation.

Sociodemographic factors negatively associated with SRT scores across both age subgroups were female sex and age, treated as a continuous measure beyond the stratification. Additional sociodemographic factors associated with lower SRT scores among participants aged <65 were annual income <$25,000 and identifying as Black or African American race. The only additional sociodemographic factor in the SRT model for participants aged ≥65 years was hispanic ethnicity, which was also associated with a lower SRT score (Figures 3A,B).

Health conditions and behaviors that were associated with worse SRT scores across both age groups were chronic headaches. Among participants aged <65 years, type 2 diabetes mellitus, osteoarthritis, COPD, hypertension, and major depressive disorder were associated with lower SRT score; conversely, colon polyps were associated with higher SRT score. Among participants aged ≥65 years, many health conditions including stroke, pulmonary embolism/deep venous thrombosis, coronary artery disease, NAFLD, peptic ulcer, and Hepatitis B were associated with lower SRT score while melanoma skin cancer and tinnitus were associated with higher SRT score.

BMI was negatively associated with SRT score in both age groups. Additional biomarkers negatively associated with SRT scores in participants <65 included HbA1c, CRP, and mean platelet volume, while cholesterol, Vitamin D, and platelet count had positive associations with SRT scores in this age group. Among participants ≥65 years, increased basophil count, neutrophil count, and glomerular filtration rate were associated with lower SRT scores, while increased urine albumin and hemoglobin were associated with higher SRT scores.

####  30-second chair stand test

**Figure 4:**
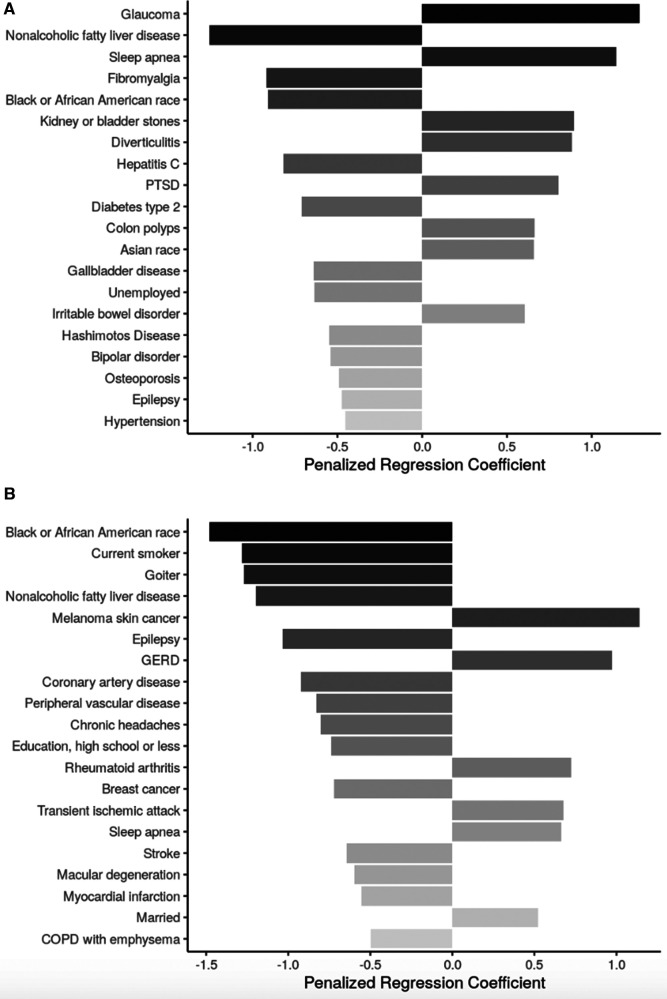
Top 20 regression coefficients of features selected from sENET regression model for 30-s chair stand test. (**A**) Participants <65 years of age (*ɑ* = 0.5, *λ* = 0.0238); (**B**): participants ≥65 years of age (*ɑ* = 0.5, *λ* = 0.0752). PTSD, posttraumatic stress disorder; GERD, gastroesophageal reflux disease; COPD, chronic obstructive pulmonary disease.

The only sociodemographic factor shared across both age subgroups in the models for the 30CST was being Black or African American (associated with lower stand count). Among adults aged <65 years, other sociodemographic characteristics in the model were Asian race (associated with higher stand count) and being unemployed (associated with lower stand count). Among adults aged ≥65 years, additional sociodemographic factors in the 30CST model were having high school or less as highest education attainment, which was associated with lower stand count, while being married was associated with higher stand count (Figures 4A,B).

Health conditions and behaviors that were associated with lower 30CST count across both age groups were NALFD and epilepsy. Sleep apnea was also present in the models for both age subgroups, but was associated with improved 30CST performance. Among participants aged <65 years, glaucoma, kidney or bladder stones, diverticulitis, PTSD, colon polyps, and IBD were also associated with a higher stand count while fibromyalgia, hepatitis C, type 2 diabetes mellitus, gallbladder disease, and Hashimoto's disease were among the conditions associated with lower stand count. Among participants aged ≥65 years, melanoma skin cancer, GERD, rheumatoid arthritis, and transient ischemic attack were associated with higher stand count while a variety of conditions, including being a current smoker, goiter, epilepsy, coronary artery disease, peripheral vascular disease, breast cancer, and stroke were associated with lower stand count.

Neither of the models for 30CST identified any biomarkers as one of the top 20 features selected.

### Sensitivity analysis results

We present results from sensitivity analyses in the Supplementary Material.

## Discussion

This study used a deeply phenotyped cohort to identify variables possibly associated with three physical performance tests. We found associations that stood out across performance tests and age-stratification, including sociodemographic characteristics, health conditions/behaviors, and biomarker values. After selecting top-five features across tests and age groups, being Black or African American was a top-five candidate feature across performance tests among participants aged <65 years, and NAFLD was a consistently top-five feature among participants aged ≥65.

In general, physical function measures were slightly higher in the PBHS cohort than age-based reference values for SLBT, SRT and 30CST in previous reports based on community-dwelling adults (primarily aged ≥60 years) and in disease-based populations. More specifically, the SLBT time was higher across both age groups compared to previously published data (20, 29). For the 30CST, the mean stand count among adults aged ≥65 was only slightly higher than a prior reference (30). Prior data are scarce for comparisons in the <65 year group, but the distributions of the 30CST results were relatively similar across age-stratified groups. While age-based median SRT values have been reported, direct comparability with our is limited (23).

### Commonalities across sociodemographic characteristics

We found several sociodemographic characteristics associated with physical performance, for example: race and ethnicity, income level, and educational attainment. These could be considered consistent with previous research, particularly if we assume that general physical activity is closely related to physical performance measures. For instance, there are prior reports of higher educational attainment associated with physically active lifestyles (31–33). Substantial physical activity disparities by race and income have also been previously reported for adolescents and young adults (34). Taken together, this research underlines the complex relationship between physical performance, sociodemographics and social determinants of health. Additional research is warranted to understand the impact, if any, of targeted physical activity interventions on the associations between physical performance and sociodemographic factors.

### Commonalities across health conditions and behaviors

Our results confirm previous findings associating reduced physical function with health status, which highlight the potential to identify subclinical disease in patients at risk. For example, the associations of both SLBT and SRT with hypertension (age <65) and with history of stroke or coronary artery disease (age ≥65) indicate a potential value in predicting cardiovascular disease conditions or events.

NAFLD was notably associated with worse performance on the SLBT, SRT, and 30CST and was a top associated factor across both age groups. NAFLD is over two-times more prevalent among adults who are overweight (75%) or obese (76%) (35). Future research could validate the role of SLBT, SRT, and 30CST in low-cost screening prior to NAFLD full diagnostic testing.

Among those aged ≥65, each of the physical performance measures was negatively associated with experiencing chronic headaches. Although previous research indicates chronic headaches tend to have negative effects on physical activity (36), it is unclear why this consistent relationship was limited to those ≥65 years-old, pending future research.

### Commonalities across biomarkers

Biomarkers associated with each of the three physical performance tests had limited commonalities across tests and age-groups. While only associated with two physical performance tests in the younger population (SLBT and SRT), CRP was shown to be negatively associated with each. CRP is known as a marker of inflammation (37), either acute due to infectious disease or chronic due to other disease states, such as atherosclerotic cardiovascular disease (38), both of which can impact physical function.

We also found a negative association between BMI and SLBT scores (in the older population) and SRT scores (in both age groups). Prior research has similarly reported on an inverse relationship between body composition and physical function measures (39–41).

### Limitations

First, this is an exploratory study, cross-sectional in nature, aimed at identifying associations without implying causality (42). Follow-up studies could determine and validate the individual diagnostic or prognostic predictive value SLBT, SRT, and 30CST related to these novel associations, but determining causal effects will require an alternative methodological approach (43). Second, while the PBHS participants are overall representative of U.S. adult age, sex, race, and ethnicity (18), recruitment sites were limited to two states (California and North Carolina) which may limit generalizability. Third, analyses were stratified into two age-based groups (<65 and ≥65 years) only, due to sample size limitations; further research is warranted to identify associations specific to more granular age subgroups.

In this study, we identified several novel salient factors with the SLBT, SRT, and 30CST when stratifying by age—many of them common across the three tests. Our results support the need for further investigation of the potential for these physical performance tests as affordable, noninvasive biomarkers of prevalent conditions and their predictive utility for incident health conditions in later life (e.g., cardiovascular disease and/or events) in community-dwelling adults. Further research would also be warranted to estimate the direction and magnitude of any causal relationships between these physical function tests and novel factors identified where biologically plausible.

## Data Availability

The de-identified Project Baseline Health Study (PBHS) data corresponding to this study are available upon request for the purpose of examining its reproducibility. Requests are subject to approval by PBHS governance. Requests to access the datasets should be directed to megankcarroll@verily.com.
